# Histological and histomorphometric effects of hyaluronic acid with or without xenograft on rat extraction socket healing

**DOI:** 10.1186/s12903-026-10120-z

**Published:** 2026-11-10

**Authors:** Aliaa A. Habib, Eman M. Salem

**Affiliations:** 1 Oral and Maxillofacial Surgery Department, Faculty of Dentistry, Pharos University in Alexandria, Alexandria, Egypt; 2 Oral Biology Department, Faculty of Dentistry, Pharos University in Alexandria, Alexandria, Egypt

**Keywords:** Hyaluronic acid, Extraction socket healing, Xenograft, Histomorphometry, Bone regeneration

## Abstract

**Background:**

Alveolar bone loss following tooth extraction remains a major clinical concern that may compromise future implant placement and prosthetic rehabilitation. Hyaluronic acid (HA), a natural component of the extracellular matrix, exhibits anti-inflammatory, angiogenic, and osteogenic properties that may enhance post-extraction healing. This experimental study evaluated the effect of HA, used alone or combined with a xenograft, on bone regeneration in extraction sockets.

**Methods:**

Twenty adult female Sprague–Dawley rats were included. Two animals served as untreated baseline histological references, while the remaining eighteen rats underwent mandibular first molar extraction and were randomly allocated into three groups (*n* = 6/group): extraction only (control), HA application, and HA combined with xenograft. Each group was further evaluated at 2, 4, and 6 weeks post-extraction, with two animals assessed at each healing interval. Histological evaluation was performed using hematoxylin and eosin (H&E) and Masson’s trichrome staining. Histomorphometric analysis quantified the percentage of newly formed bone area within standardized regions of interest using calibrated ImageJ software. Differences among treatment groups were analyzed separately at each healing interval using one-way ANOVA or the Kruskal–Wallis test, as appropriate, followed by appropriate post-hoc multiple comparisons. Statistical significance was set at *p* < 0.05.

**Results:**

Both HA-treated groups demonstrated significantly greater bone formation compared with extraction alone at all healing intervals (*p* < 0.001). Histological assessment revealed reduced inflammatory infiltrate and more organized trabecular architecture in HA-treated sockets. Masson’s trichrome staining demonstrated increased collagen deposition and matrix organization, particularly in the HA + xenograft group. Although the combination therapy demonstrated numerically higher bone area values throughout the healing period, no statistically significant difference was observed between HA alone and HA combined with xenograft (*p* > 0.05).

**Conclusion:**

Within the limitations of this exploratory pilot animal study, HA significantly enhanced extraction socket healing by promoting new bone formation, improving trabecular organization, and increasing collagen deposition compared with spontaneous healing. The addition of xenograft resulted in slightly higher histological and histomorphometric values; however, it did not result in statistically superior bone formation compared with HA alone within the experimental timeframe. These findings suggest that HA may represent a promising adjunctive biomaterial for alveolar ridge preservation. Nevertheless, larger preclinical studies and well-designed randomized clinical trials are required to validate these findings and determine their clinical applicability.

**Clinical relevance:**

The adjunctive application of hyaluronic acid (HA), with or without bone substitute materials, may enhance post-extraction bone quality and accelerate healing dynamics. This could support more predictable ridge preservation outcomes and potentially facilitate early implant placement by improving the quantity and quality of newly formed bone. Therefore, HA may represent a clinically relevant biomaterial in contemporary alveolar ridge preservation protocols.

**Graphical Abstract:**

**Figure:**
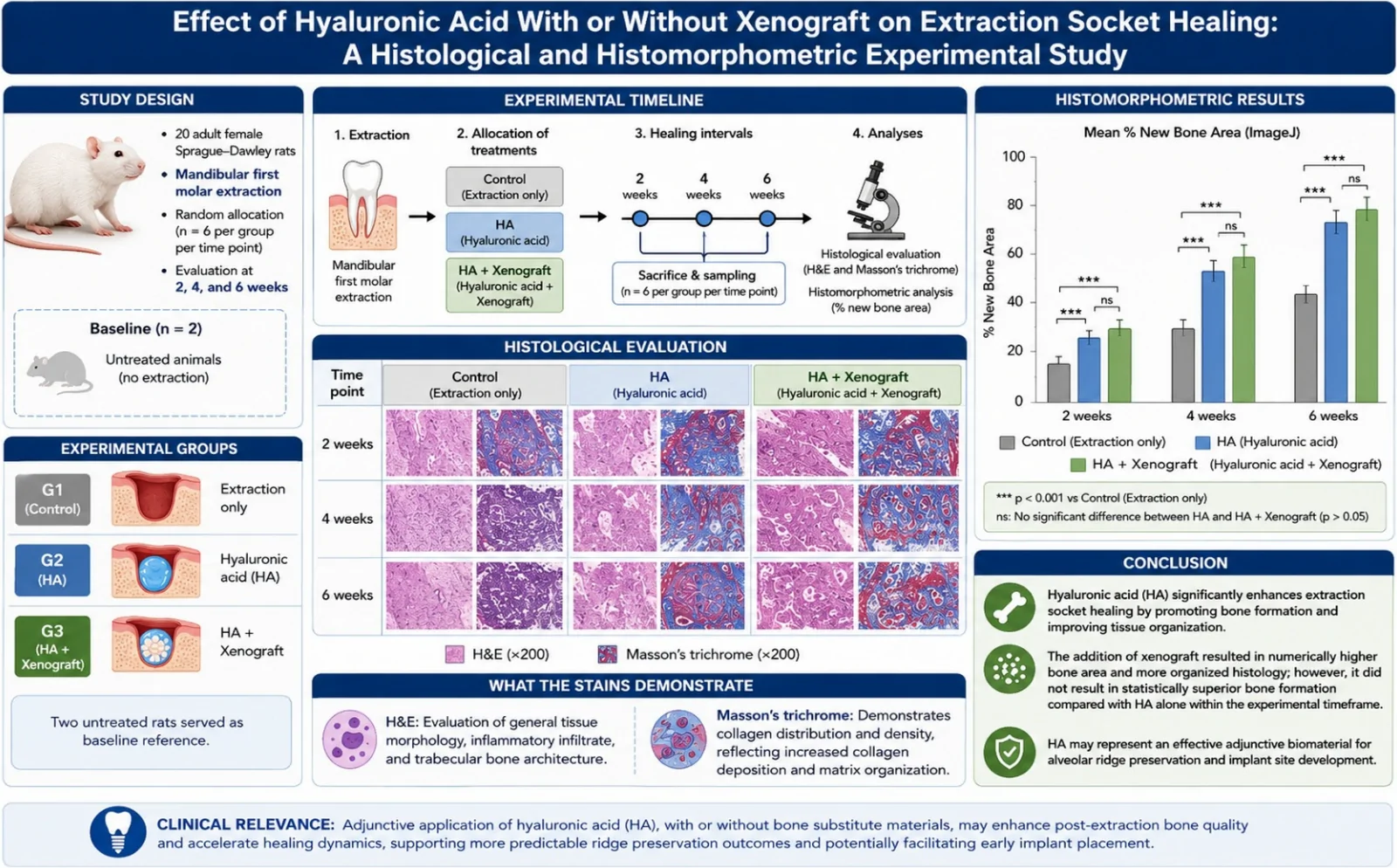

## Introduction

Tooth extraction initiates a complex biological cascade that ultimately leads to healing of the extraction socket and remodeling of the surrounding alveolar bone. Although socket healing is a physiological process, considerable horizontal and vertical ridge resorption occurs during the first few months after extraction, resulting in significant loss of alveolar bone volume and soft tissue architecture [1–4]. These dimensional changes may compromise future implant placement, prosthetic rehabilitation, and the overall esthetic outcome, particularly in the anterior region. Consequently, alveolar ridge preservation (ARP) has become an important therapeutic approach aimed at minimizing post-extraction tissue loss and maintaining favorable ridge dimensions for subsequent implant therapy [5–7].

A wide variety of biomaterials have been investigated for ARP, including autogenous, allogeneic, xenogeneic, and alloplastic bone substitutes. Among these, bovine-derived xenografts are widely used because of their osteoconductive properties and their ability to provide a scaffold that supports new bone formation and helps maintain the dimensions of the healing site [8–10]. However, xenografts primarily function as osteoconductive scaffolds and do not necessarily provide direct biological modulation of the inflammatory and reparative processes. Therefore, increasing attention has been directed toward combining osteoconductive graft materials with biologically active agents that may enhance the biological events associated with tissue repair [11–13].

Hyaluronic acid (HA) is a naturally occurring, non-sulfated glycosaminoglycan that represents a major component of the extracellular matrix in connective tissues. Beyond its structural role, HA has been implicated in several processes involved in wound healing and tissue regeneration, including modulation of inflammation, cell migration, extracellular matrix organization, and tissue remodeling [14–16]. The biological activity of HA is influenced by several factors, including its molecular weight, concentration, formulation, and degree of cross-linking. High-molecular-weight HA has generally been associated with anti-inflammatory and immunomodulatory effects, whereas lower molecular weight fragments may exhibit different biological activities, including effects on cellular proliferation and angiogenic responses [14, 15]. Therefore, different HA formulations may produce different biological responses, highlighting the importance of clearly identifying the characteristics of the material used in experimental and clinical studies.

In recent years, increasing attention has been directed toward the combined use of HA with bone graft substitutes in regenerative procedures. The rationale for this combination is that HA may provide a biologically favorable environment, while the xenograft acts primarily as an osteoconductive scaffold. Clinical studies have reported encouraging outcomes following the use of HA, alone or in combination with bovine-derived xenografts, for alveolar ridge preservation, including histological and histomorphometric assessments [17–19]. Recent systematic and narrative reviews have also suggested that HA may represent a promising adjunctive biomaterial for alveolar ridge preservation; however, substantial heterogeneity remains among published studies with respect to HA formulations, graft materials, study designs, and outcome assessment methods [20, 21].

Despite the growing body of evidence supporting the regenerative potential of HA, important knowledge gaps remain. Most previous investigations have evaluated HA alone or HA combined with graft materials independently, whereas only a limited number of preclinical studies have directly compared the biological effects of HA alone with those of HA combined with a xenogeneic bone substitute using standardized histological and histomorphometric analyses within the same experimental model. Consequently, it remains unclear whether the addition of a xenogeneic bone substitute provides measurable regenerative benefits beyond those achieved by HA alone during the early stages of extraction socket healing [17–19].

Histological and histomorphometric analyses provide complementary approaches for evaluating tissue morphology and quantifying bone formation in preclinical models. Standardized histological preparation, staining, image acquisition, and digital image analysis allow objective assessment and comparison of regenerative outcomes [9, 11]. In particular, histomorphometric analysis enables quantitative assessment of the proportion of newly formed bone within standardized regions of interest, providing an objective measure of regenerative changes during socket healing.

Therefore, the present experimental study aimed to evaluate the effect of hyaluronic acid, used alone or in combination with a bovine-derived xenogeneic bone graft, on extraction socket healing in a rat model using standardized histological and histomorphometric analyses. We hypothesized that adjunctive application of HA would enhance bone regeneration compared with spontaneous socket healing and that the addition of a xenograft might further improve the quality and organization of newly formed bone during the healing process.

## Materials and methods

### Study design, experimental animals, and ethical approval

This exploratory pilot animal study was conducted in accordance with the ARRIVE 2.0 guidelines and was approved by the Institutional Animal Care and Use Committee (IACUC), Pharos University in Alexandria, Egypt (Approval No. PUA02202410273281; approval date: 25 October 2024). All experimental procedures complied with the National Institutes of Health Guide for the Care and Use of Laboratory Animals and institutional ethical regulations.

Twenty healthy adult female Sprague–Dawley rats weighing 200–250 g were included in this study. Animals were obtained from the Animal House, Faculty of Dentistry, Pharos University in Alexandria, Egypt. They were housed under standardized laboratory conditions at 22 ± 2 °C with a relative humidity of 50–60% under a 12-hour light/dark cycle. Standard laboratory chow and water were provided *ad libitum*. Animals were acclimatized for one week before the surgical procedures.

Two animals served as untreated baseline histological references and were not included in the statistical analysis. The remaining eighteen animals underwent mandibular first molar extraction and were randomly allocated into three experimental groups (*n* = 6 per group) using a computer-generated randomization sequence.

Each experimental group was further subdivided according to the healing interval (2, 4, and 6 weeks), with two animals evaluated at each time point. Histological and histomorphometric analyses were performed using the extraction sockets obtained from these animals.

The sample size was determined based on the exploratory and pilot nature of the study, ethical considerations regarding the use of experimental animals, and practical feasibility. Six animals were allocated to each treatment group, with two animals assessed at each healing interval (2, 4, and 6 weeks). Given the limited number of animals at each time point, this study was considered a pilot preclinical investigation intended to provide preliminary histological and histomorphometric evidence and to inform future adequately powered studies.

### Experimental groups

- Group I (Control): Extraction sockets were left to heal spontaneously without any intervention
- Group II (HA): Extraction sockets were treated with high-molecular-weight hyaluronic acid (HyalOne^®^, Fidia Farmaceutici S.p.A., Abano Terme, Italy)
- Group III (HA + Xenograft): Extraction sockets were treated with deproteinized bovine bone mineral (OneXeno Graft^®^, OneGraft, Germany), followed by the application of high-molecular-weight hyaluronic acid

Animals were euthanized at 2, 4, and 6 weeks after tooth extraction for histological and histomorphometric evaluation.

### Biomaterials

The hyaluronic acid (HA) used in this study was a high-molecular-weight sodium hyaluronate gel (HyalOne^®^, Fidia Farmaceutici S.p.A., Abano Terme, Italy), containing 60 mg/4 mL (1.5%) sodium hyaluronate produced by bacterial fermentation. The material was supplied as a pre-filled sterile syringe and was applied directly into the extraction socket according to the manufacturer’s instructions. The xenogeneic bone graft used was deproteinized bovine bone mineral (DBBM) (OneXeno Graft^®^, OneGraft, Germany). The graft particles measured < 2.0 mm and were placed into the extraction socket before the application of hyaluronic acid.

In the HA group, HA was applied directly to the extraction socket, whereas in the HA + xenograft group, the xenograft was first placed in the socket and was subsequently covered with HA. The exact amount of HA and xenograft applied per socket was not prospectively quantified/recorded. The HA gel was applied according to the manufacturer’s instructions, while the xenograft was placed to fill the extraction socket as described in the experimental protocol. We have clarified this point in the Methods and acknowledged the lack of precise per-socket quantification as a methodological limitation.

### Surgical procedure

All surgical procedures were performed by the same experienced operator under aseptic conditions.

General anesthesia was induced by intraperitoneal administration of ketamine hydrochloride (90 mg/kg) and xylazine hydrochloride (10 mg/kg). Adequate anesthesia was confirmed by the absence of the pedal withdrawal reflex.

The surgical field was disinfected using povidone–iodine solution. Mandibular first molars were atraumatically extracted using micro-forceps under magnification to minimize trauma to the socket walls. Following extraction, the sockets were gently irrigated with sterile saline to remove blood clots and debris.

In Group II, the extraction sockets were filled with hyaluronic acid using a sterile syringe according to the manufacturer’s instructions. In Group III, **0.1 g (100 mg) of xenogeneic bone graft** was first placed into each extraction socket, followed by application of hyaluronic acid to cover the graft material. This layered approach was used to maintain the xenograft within the socket as an osteoconductive scaffold while allowing HA to be applied as a separate adjunctive biomaterial rather than being mechanically mixed with the graft. This application sequence reflected the experimental protocol used in the present study. Hemostasis was achieved using sterile gauze compression without suturing.

### Postoperative care

Postoperative analgesia was provided using meloxicam (1–2 mg/kg, subcutaneously) once daily for three consecutive days. Animals were monitored daily for general health, wound healing, signs of infection, and behavioral changes throughout the experimental period.

### Specimen collection

At the predetermined healing intervals (2, 4, and 6 weeks), animals were euthanized by anesthetic overdose followed by cervical dislocation. Mandibular specimens containing the extraction sockets were harvested and immediately fixed in 10% neutral buffered formalin. The specimens were oriented and sectioned in a standardized buccolingual plane passing through the center of the extraction socket to ensure consistency among all samples.

### Histological processing and staining

Specimens were fixed in 10% neutral buffered formalin for 48 h and decalcified using an ethylenediaminetetraacetic acid (EDTA)-based solution. Complete decalcification was confirmed before routine tissue processing. Following dehydration through ascending grades of ethanol, the specimens were embedded in paraffin wax. Serial longitudinal buccolingual Sect.  (5 μm thick) passing through the center of the extraction socket were prepared using a rotary microtome.

The tissue sections were stained with:

- Hematoxylin and eosin (H&E) for general histological evaluation.
- Masson’s trichrome stain for the assessment of collagen deposition.

All photomicrographs were captured using identical microscope settings under standardized magnifications. Histological evaluation and histomorphometric measurements were performed by **a blinded examiner** who remained unaware of the treatment allocation throughout the evaluation process, thereby minimizing observer bias.

### Histomorphometric analysis

Histomorphometric analysis was performed using ImageJ software (National Institutes of Health, Bethesda, MD, USA). A standardized region of interest (ROI) corresponding to the entire extraction socket was selected for all specimens. The percentage of newly formed bone area (%) was calculated relative to the total socket area. All measurements were performed under identical magnification and image acquisition settings to ensure consistency. Histological evaluation and histomorphometric measurements were performed by an examiner blinded to the experimental groups to minimize observer bias.

### Statistical analysis

All statistical analyses were performed using IBM SPSS Statistics version 25.0 (IBM Corp., Armonk, NY, USA). The primary quantitative outcome was the percentage of newly formed bone area within a standardized region of interest (ROI) corresponding to the extraction socket. Histomorphometric measurements were obtained from calibrated digital images using ImageJ software (National Institutes of Health, USA).

Data were inspected for outliers and assessed for normality using the Shapiro–Wilk test. Homogeneity of variances among treatment groups was evaluated using Levene’s test. Because different animals were evaluated at each healing interval, observations obtained at 2, 4, and 6 weeks were considered independent. Accordingly, repeated-measures and mixed-effects analyses were not performed.

At each healing interval (2, 4, and 6 weeks), differences in newly formed bone area among the three treatment groups (control, HA, and HA + xenograft) were analyzed separately using one-way analysis of variance (ANOVA) when the assumptions of normality and homogeneity of variance were satisfied. When a statistically significant overall group effect was detected, post-hoc pairwise comparisons were performed using the Tukey HSD test to identify specific between-group differences.

For comparisons among the three independent healing intervals within each treatment group, separate one-way ANOVA analyses were performed when the parametric assumptions were satisfied, followed by Tukey HSD post-hoc comparisons when appropriate. These analyses were interpreted as comparisons between independent animals evaluated at different time points rather than as longitudinal changes within the same animals.

When the assumptions required for one-way ANOVA were not satisfied, the Kruskal–Wallis test was used, followed by Dunn’s multiple-comparison test with Bonferroni adjustment for post-hoc pairwise comparisons.

The histomorphometric analysis was performed using standardized ROI boundaries, calibrated with a stage micrometer. Thresholding and segmentation procedures were kept consistent across all specimens. All measurements were performed by an examiner blinded to the experimental groups. To assess measurement reproducibility, 20% of the specimens were independently re-evaluated by a second examiner.

Results are presented as mean ± standard deviation (SD), together with 95% confidence intervals (CI) where appropriate. Statistical significance was set at *p* < 0.05. Given the limited number of animals available at each time point, the findings were considered exploratory and should be interpreted with appropriate caution.

## Results

### Histological findings

#### Week 2

##### Control group

**Fig. 1:**
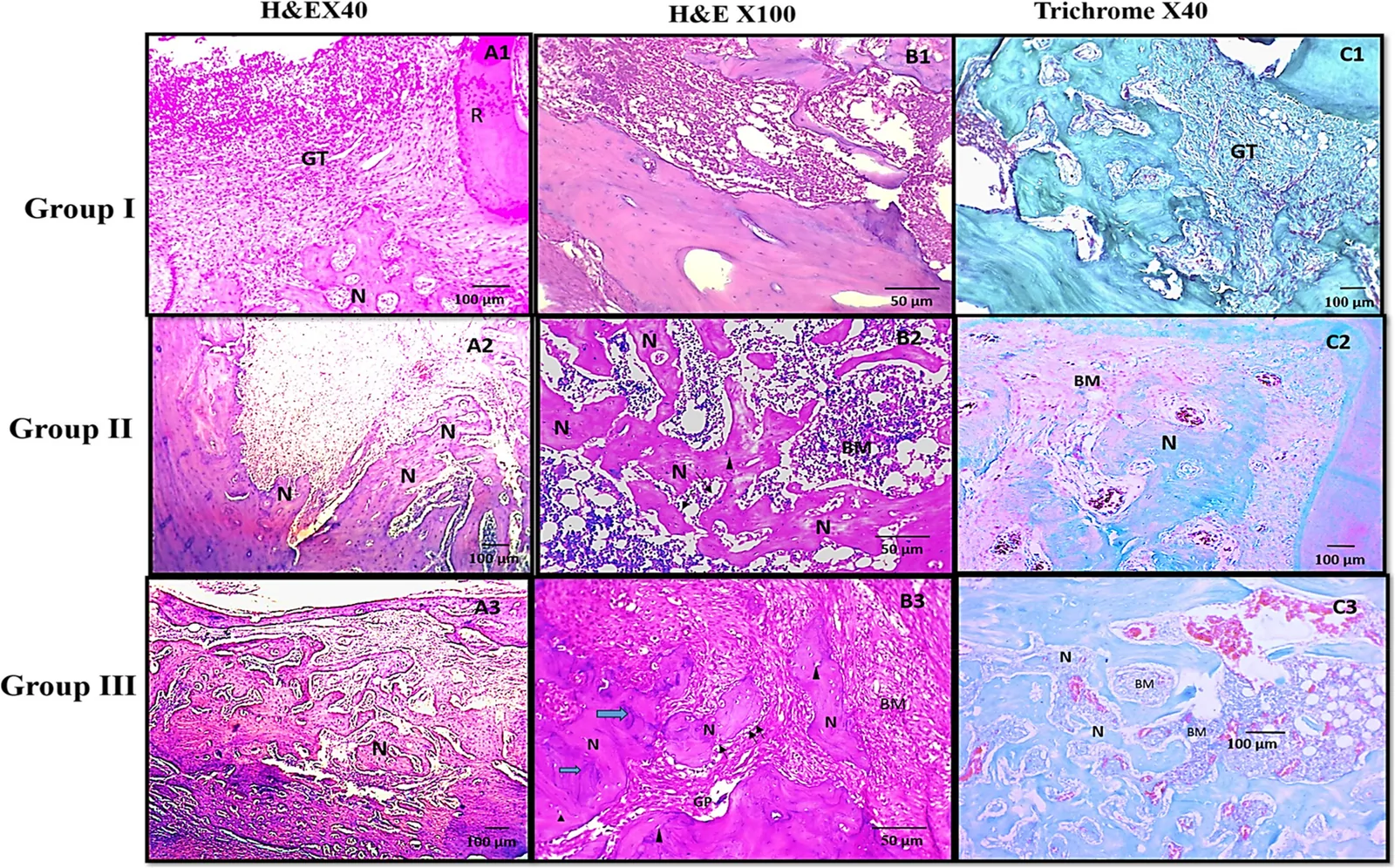
Representative histological sections illustrating extraction socket healing at 2 weeks in the control, hyaluronic acid (HA), and HA + xenograft groups stained with hematoxylin and eosin (H&E) and Masson’s trichrome. **A1**–**A3** (Control group): H&E sections demonstrate abundant granulation tissue (GT), inflammatory cell infiltration, and limited newly formed bone (N). Masson’s trichrome staining shows sparse collagen deposition. **B1**–**B3** (HA group): Higher-magnification H&E sections demonstrate newly formed woven bone characterized by osteoblast lining (thin arrows), osteocytes within bone lacunae (arrowheads). Masson’s trichrome staining demonstrates increased collagen deposition compared with the control group. **C1**–**C3** (HA + xenograft group): H&E sections demonstrate extensive newly formed bone with improved trabecular organization characterized by osteoblast lining (thin arrows), osteocytes within bone lacunae (arrowheads).and reversal lines (blue arrows), indicating active bone remodeling Masson’s trichrome staining reveals dense collagen deposition and well-organized connective tissue. Abbreviations: GT, granulation tissue; N, newly formed bone; R, root. Symbols: Thin arrows indicate osteoblasts; arrowheads indicate osteocytes; blue arrows indicate reversal lines. Original magnifications: **A1**–**A3** and **C1**–**C3**, ×40; **B1**–**B3**, ×100. Scale bars are shown in each panel

At 2 weeks, the extraction sockets of the control group were predominantly occupied by granulation tissue (GT) with dense inflammatory cell infiltration. Limited newly formed woven bone (N) was observed along the socket walls. Masson’s trichrome staining demonstrated sparse collagen deposition within the healing socket (Fig. 1A1–A3).

##### HA group

The HA-treated group exhibited a greater amount of newly formed woven bone compared with the control group. The trabeculae appeared thicker and more interconnected, with reduced inflammatory cell infiltration. Higher-magnification sections demonstrated osteoblast lining along the trabecular surfaces and osteocytes embedded within bone lacunae, indicating active bone formation. Masson’s trichrome staining showed increased collagen deposition compared with the control group (Fig. 1B1–B3).

##### HA + xenograft group

The HA + xenograft group demonstrated abundant newly formed bone with improved trabecular organization compared with the control group. Bone formation was observed around the grafted area with minimal inflammatory infiltrate. Masson’s trichrome staining demonstrated dense collagen deposition and well-organized connective tissue matrix (Fig. 1C1–C3).

#### Week 4

##### Control group

**Fig. 2:**
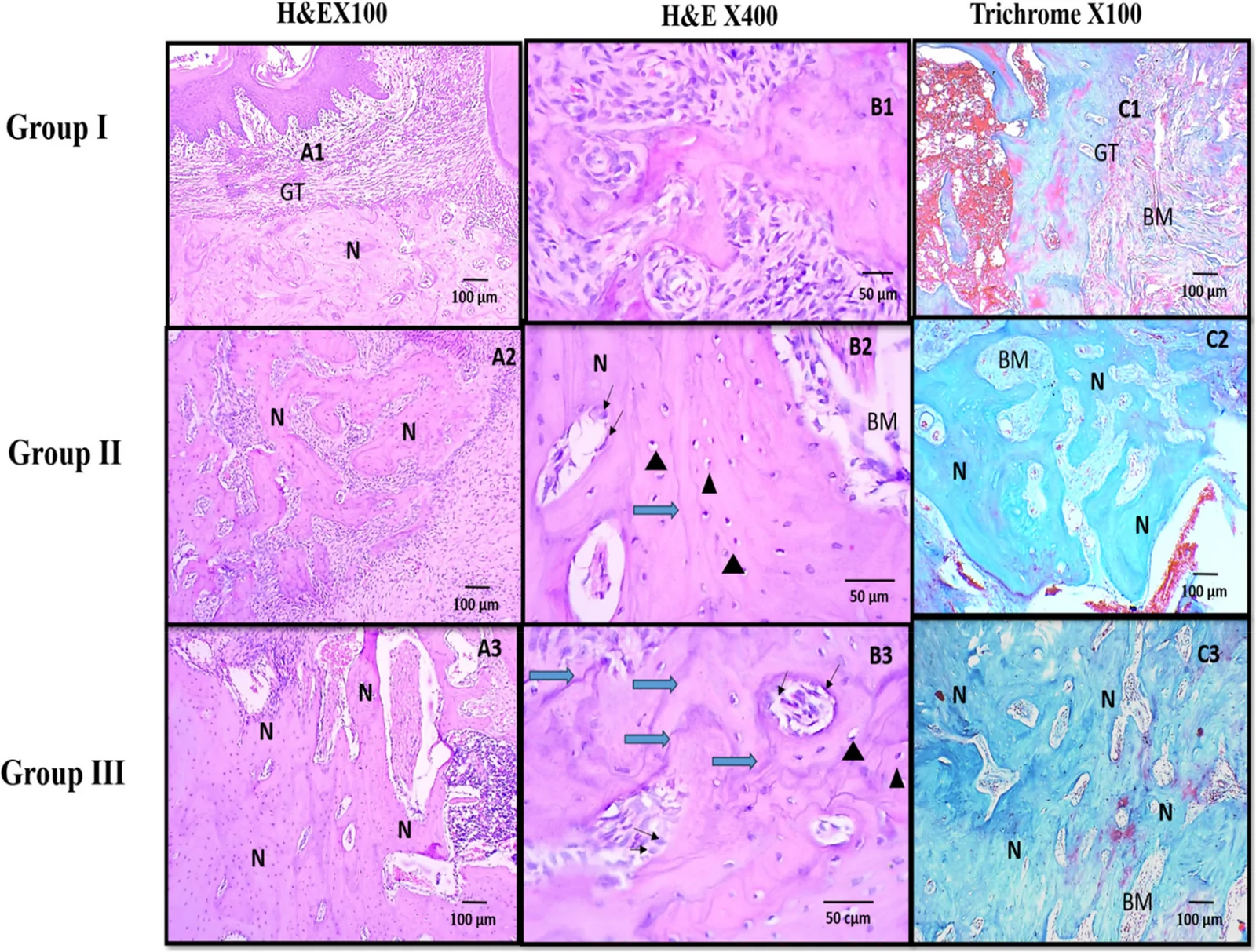
Representative histological sections illustrating extraction socket healing at 4 weeks in the control, hyaluronic acid (HA), and HA + xenograft groups stained with hematoxylin and eosin (H&E) and Masson's trichrome. **A1**–**A3** (Control group): H&E sections demonstrate persistent granulation tissue (GT) with moderate newly formed bone (N) and incomplete trabecular organization. Masson's trichrome staining reveals relatively sparse collagen deposition and prominent bone marrow spaces (BM). **B1**–**B3** (HA group): H&E sections demonstrate active bone remodeling with well-developed newly formed bone trabeculae. Higher magnification (**B2** and **B3**) shows osteoblast lining (thin arrows), osteocytes within bone lacunae (arrowheads), and reversal lines (blue arrows), indicating ongoing bone remodeling. Bone marrow spaces (BM) are evident between the trabeculae. Masson's trichrome staining demonstrates increased collagen deposition compared with the control group. **C1**–**C3** (HA + xenograft group): H&E and Masson's trichrome sections demonstrate abundant newly formed bone (N) with well-organized trabecular architecture, prominent bone marrow spaces (BM), and dense collagen deposition, indicating advanced socket healing. Abbreviations: GT, granulation tissue; N, newly formed bone; BM, bone marrow spacesزSymbols: Thin arrows indicate osteoblasts; arrowheads indicate osteocytes; blue arrows indicate reversal lines. Original magnifications: **A1**–**C1** and **A3**–**C3**, ×100; **A2**–**C2**, ×400. Scale bars: 100 μm (×100) and 50 μm (×400)

At 4 weeks, the control group exhibited moderate progression of bone healing. Newly formed trabeculae occupied a larger portion of the socket; however, persistent granulation tissue and irregular trabecular organization were still evident. Masson’s trichrome staining demonstrated moderate collagen deposition and relatively wide bone marrow spaces (Fig. 2A1–A3).

##### HA group

The HA-treated sockets demonstrated more extensive newly formed bone with thicker and better-organized trabeculae. Higher-magnification sections showed osteoblast lining, numerous osteocytes within bone lacunae, and reversal lines, indicating active bone remodeling. Bone marrow spaces (BM) were observed between the trabeculae. Masson’s trichrome staining demonstrated increased collagen deposition compared with the control group (Fig. 2B1–B3).

##### HA + xenograft group

The HA + xenograft group demonstrated abundant newly formed bone with well-organized trabecular architecture and clearly defined bone marrow spaces. Collagen deposition appeared denser than in the control group. The regenerated bone exhibited a more organized appearance throughout the extraction socket (Fig. 2C1–C3).

#### Week 6

##### Control group

**Fig. 3:**
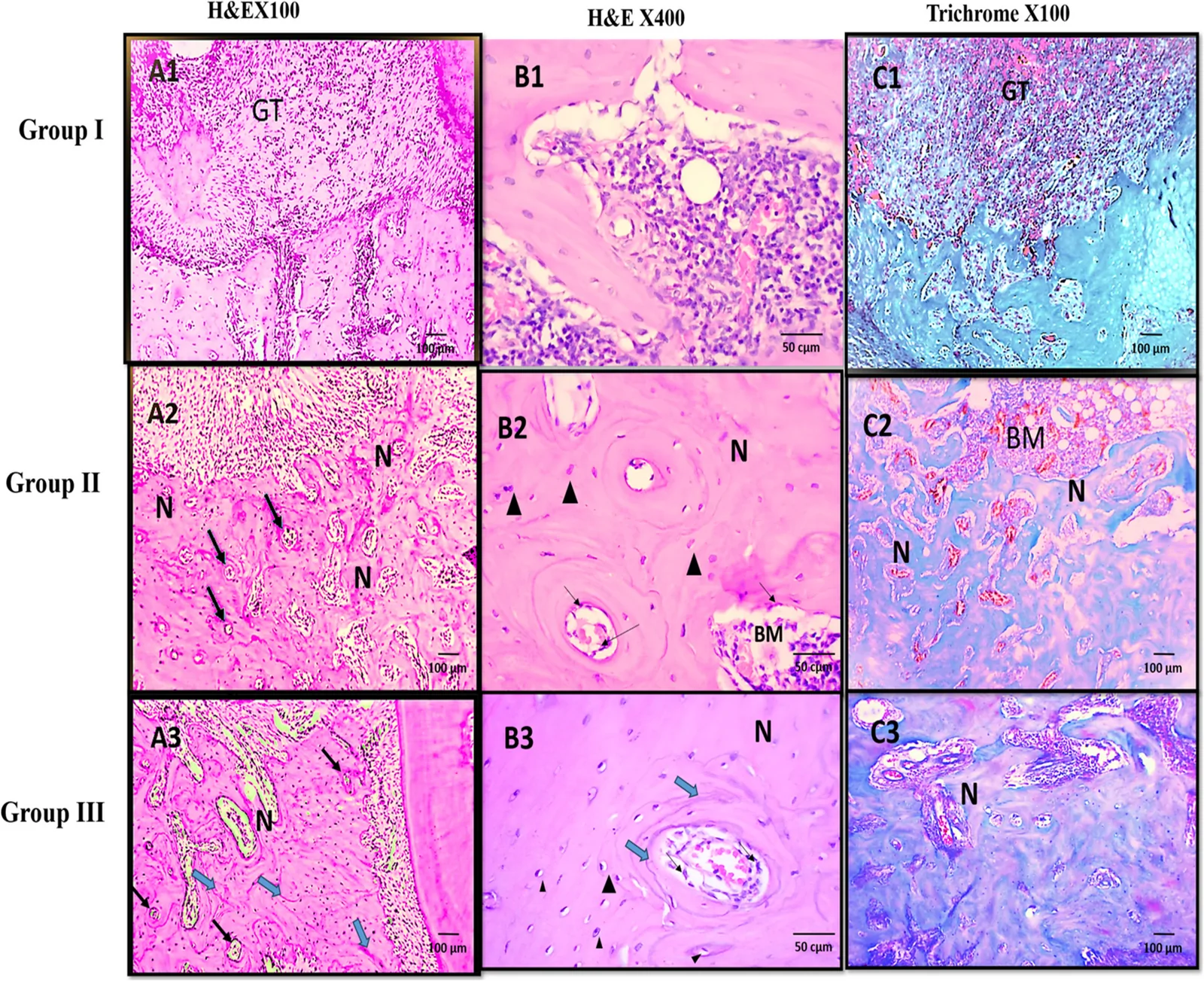
Representative histological sections illustrating extraction socket healing at 6 weeks in the control, hyaluronic acid (HA), and HA + xenograft groups stained with hematoxylin and eosin (H&E) and Masson’s trichrome.**A1–A3 **(Control group): H&E sections demonstrate persistent granulation tissue (GT) with moderate newly formed bone (N). Higher magnification shows ongoing bone remodeling with limited maturation. Masson’s trichrome staining demonstrates moderate collagen deposition. **B1–B3 **(HA group): H&E sections demonstrate advanced bone remodeling with abundant newly formed bone (N). Higher-magnification images reveal osteocytes within bone lacunae (arrowheads), well-developed Haversian systems (bold arrows), and reversal lines (blue arrows), indicating progressive maturation and remodeling of the newly formed bone. Bone marrow spaces (BM) are present between the trabeculae. Masson’s trichrome staining demonstrates dense collagen deposition and a more organized bone matrix. **C1–C3 **(HA + xenograft group): H&E and Masson’s trichrome sections demonstrate abundant, well-organized newly formed bone (N) with prominent bone marrow spaces (BM) and dense collagen deposition, consistent with advanced socket healing. Higher-magnification images (**C2** ) reveal well-organized trabecular bone containing numerous osteocytes within lacunae, osteoblast lining well-defined bone marrow (BM) cavities, indicating active bone remodeling and progressive maturation of the regenerated bone. Abbreviations: GT, granulation tissue; N, newly formed bone; BM, bone marrow spaces.Symbols: Thin arrows indicate osteoblasts; arrowheads indicate osteocytes; blue arrows indicate reversal lines; bold arrows indicate Haversian systems. Original magnifications: **A1**–**C1** and **A3**–**C3**, ×100; **A2**–**C2**, ×400. Scale bars: 100 μm (×100)

At 6 weeks, the control group demonstrated further progression of bone formation with increased trabecular thickness; however, residual granulation tissue remained evident in some areas. Masson’s trichrome staining showed moderate collagen deposition within the healing socket (Fig. 3A1–A3).

##### HA group

The HA-treated group exhibited advanced bone remodeling characterized by abundant newly formed bone with increased trabecular thickness. Higher-magnification sections demonstrated numerous osteocytes within bone lacunae, osteoblast lining, well-developed Haversian systems, and reversal lines, indicating ongoing maturation and remodeling of the regenerated bone. Bone marrow spaces were present between the trabeculae. Masson’s trichrome staining demonstrated dense collagen deposition and a well-organized bone matrix (Fig. 3B1–B3).

##### HA + xenograft group

The HA + xenograft group exhibited the most organized histological appearance among all groups. Extensive newly formed bone occupied the extraction socket with well-developed trabecular architecture and prominent bone marrow cavities. Higher-magnification sections demonstrated numerous osteocytes within bone lacunae, osteoblast lining along the trabecular surfaces, and well-defined bone marrow spaces, indicating advanced remodeling of the regenerated bone. Masson’s trichrome staining revealed dense collagen deposition throughout the regenerated tissue (Fig. 3C1–C3). 

### Histomorphometric findings

**Fig. 4:**
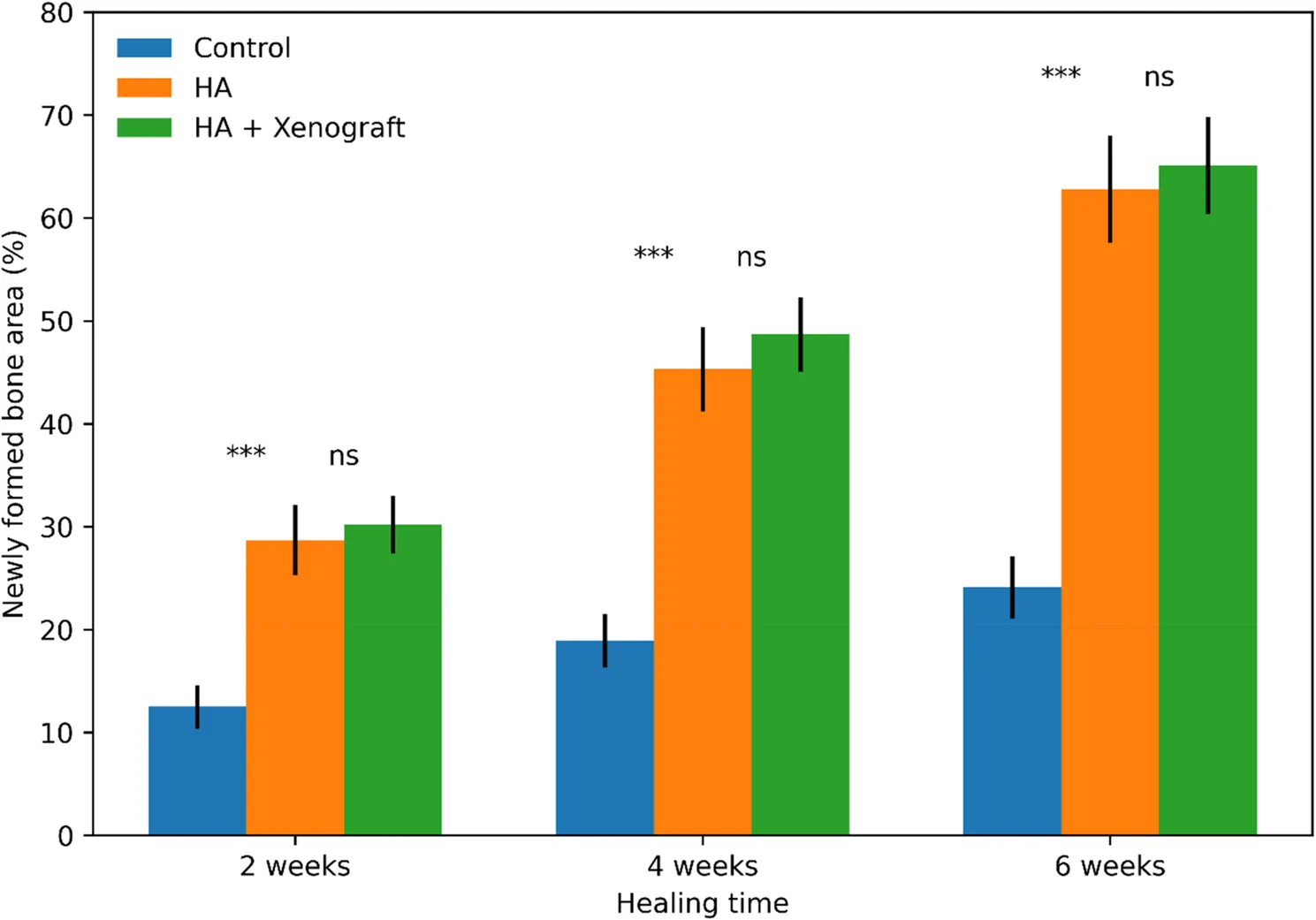
Histomorphometric analysis showing the percentage of newly formed bone area (%) in the control, HA, and HA + xenograft groups at 2, 4, and 6 weeks. Bars represent mean ± SD. Statistical significance is indicated as ***p < 0.001 versus the control group, whereas no significant difference was detected between the HA and HA + xenograft groups (ns, *p* > 0.05)

Histomorphometric analysis demonstrated a progressive increase in the percentage of newly formed bone area in all experimental groups throughout the healing period (Table 1; Fig. 4).

**Table 1:** Descriptive statistics

Time (weeks)	Control (%)	HA (%)	HA + Xenograft (%)
2 weeks	12.5 ± 2.1	28.7 ± 3.4	30.2 ± 2.8
4 weeks	18.9 ± 2.6	45.3 ± 4.1	48.7 ± 3.6
6 weeks	24.1 ± 3.0	62.8 ± 5.2	65.1 ± 4.7
Effect	*F *value	*p* value	Partial η²
Time	347.262	< 0.001	0.943
Group	1613.829	< 0.001	0.994
Time × Group	87.893	< 0.001	0.893

The percentage of newly formed bone area was quantified within a standardized region of interest (ROI) encompassing the extraction socket. Newly formed bone was identified histologically by the presence of mineralized trabecular bone containing osteocytes within lacunae and, where evident, an osteoblastic lining. The area of newly formed bone was expressed as a percentage of the total socket area. Pre-existing native bone at the socket margins was excluded from the measurement whenever it could be clearly distinguished from newly regenerated trabecular bone. In the HA + xenograft group, residual xenograft particles were also excluded from the newly formed bone area. The same magnification, image-acquisition settings, calibration, and ROI criteria were applied to all specimens.

At 2 weeks, the mean newly formed bone area was 12.5 ± 2.1% in the control group, 28.7 ± 3.4% in the HA group, and 30.2 ± 2.8% in the HA + xenograft group. Both HA-treated groups showed greater newly formed bone area than the control group (*p* < 0.001). Although the HA + xenograft group showed a slightly higher mean value than the HA group, the difference between the two treatment groups was not statistically significant (*p* > 0.05).

At 4 weeks, the mean newly formed bone area was 18.9 ± 2.6% in the control group, 45.3 ± 4.1% in the HA group, and 48.7 ± 3.6% in the HA + xenograft group. Both HA-treated groups demonstrated greater newly formed bone area than the control group (*p* < 0.001), whereas the difference between the HA and HA + xenograft groups was not statistically significant (*p* > 0.05).

At 6 weeks, the mean newly formed bone area reached 24.1 ± 3.0% in the control group, 62.8 ± 5.2% in the HA group, and 65.1 ± 4.7% in the HA + xenograft group. Both treatment groups demonstrated greater newly formed bone area than the control group (*p* < 0.001). The HA + xenograft group showed the highest mean value; however, the difference compared with the HA group was not statistically significant (*p* > 0.05).

Overall, the histomorphometric findings demonstrated greater newly formed bone area in both HA-treated groups compared with the untreated control group at all evaluated healing intervals. Although the HA + xenograft group consistently showed the highest mean values, its difference from HA alone did not reach statistical significance. Comparisons among healing intervals were considered comparisons between independent animals because different animals were evaluated at each time point. Given the limited number of animals per time point, these findings should be interpreted as exploratory.

## Discussion

The present exploratory pilot study evaluated the effect of high-molecular-weight hyaluronic acid (HA), used alone or in combination with a bovine-derived xenogeneic bone graft, on extraction socket healing using histological and histomorphometric analyses. The findings demonstrated that both HA-treated groups exhibited significantly greater new bone formation than spontaneous healing at all healing intervals. Histologically, HA-treated sockets showed earlier organization of granulation tissue, reduced inflammatory cell infiltration, enhanced trabecular bone formation, and increased collagen deposition. Although the HA + xenograft group consistently demonstrated slightly higher mean values than HA alone, these differences did not reach statistical significance.

Extraction socket healing is a dynamic biological process involving inflammation, angiogenesis, extracellular matrix remodeling, and progressive bone formation. Hyaluronic acid has received increasing attention because of its multiple biological functions during tissue repair. High-molecular-weight HA contributes to wound healing by maintaining tissue hydration, regulating inflammatory responses, enhancing fibroblast proliferation, stimulating angiogenesis, and promoting osteoblast differentiation through interactions with CD44 receptors and extracellular matrix components [22]. These biological properties create a favorable microenvironment that accelerates tissue repair and bone regeneration, which is consistent with the histological findings observed in the present study.

At the early healing stage (2 weeks), untreated sockets demonstrated abundant granulation tissue associated with inflammatory cell infiltration and only limited peripheral woven bone formation. In contrast, HA-treated groups exhibited reduced inflammatory infiltration, increased vascularization, and more extensive newly formed trabecular bone. These findings agree with previous experimental studies demonstrating that HA modulates the inflammatory phase and promotes early osteogenesis during extraction socket healing [12].

By four weeks, both HA-treated groups exhibited progressive maturation of newly formed trabeculae with increased bone marrow formation, abundant osteocytes within mineralized bone, and active osteoblast lining. The presence of distinct reversal lines indicated ongoing bone remodeling. These findings suggest that HA not only accelerates early wound healing but also supports subsequent bone remodeling and structural organization throughout the reparative phase [13].

At six weeks, bone remodeling was more advanced in both treatment groups. Histological examination demonstrated thick interconnected trabeculae, well-developed lamellar bone architecture, numerous osteocytes embedded within the mineralized bone matrix, osteoblast-lined marrow cavities, and organized Haversian systems, consistent with advanced remodeling compared with untreated controls. Masson’s trichrome staining also demonstrated dense collagen deposition throughout the regenerated bone matrix. However, because Masson’s trichrome primarily demonstrates collagen distribution and density rather than collagen maturation itself, these findings should be interpreted as evidence of increased collagen deposition and matrix organization rather than direct confirmation of collagen maturation.

The addition of xenogeneic bone graft resulted in consistently higher mean percentages of newly formed bone compared with HA alone; however, these differences were not statistically significant. Several factors may explain this observation. First, HA itself possesses considerable regenerative potential that may have masked the additional osteoconductive contribution of the xenograft during the relatively short healing period. Second, deproteinized bovine bone mineral primarily functions as a passive osteoconductive scaffold rather than an osteoinductive material. Consequently, its regenerative advantage may become more evident during longer observation periods or when evaluated using three-dimensional imaging modalities such as micro-computed tomography [11, 15].

The present findings are generally consistent with previous clinical investigations evaluating HA in alveolar ridge preservation. Eeckhout et al. reported that adjunctive HA application improved soft tissue healing following extraction while maintaining favorable socket healing outcomes [14]. Similarly, Husseini et al. demonstrated improved clinical and radiographic healing when cross-linked HA was combined with demineralized bovine bone mineral during alveolar ridge preservation. More recently, Abaza et al. reported favorable histological and histomorphometric outcomes following the use of bovine-derived xenograft combined with HA in human extraction sockets, supporting the regenerative potential of this therapeutic approach [23]. Furthermore, the recent systematic review by Ronsivalle et al. concluded that HA is a promising adjunctive biomaterial for alveolar ridge preservation because of its anti-inflammatory, angiogenic, and osteopromotive properties, while emphasizing the need for additional high-quality experimental and clinical studies to strengthen the available evidence. Although our results agree with these studies regarding the beneficial role of HA, the absence of statistically significant differences between HA alone and HA combined with xenograft in the present study may be attributed to differences in HA formulation, graft characteristics, animal model, healing duration, sample size, and outcome assessment methodology.

Unlike previous studies that mainly focused on clinical, radiographic, or histological evaluation of individual treatment modalities, the present study directly compared spontaneous socket healing, HA alone, and HA combined with xenograft under identical experimental conditions using both qualitative histological assessment and quantitative histomorphometric analysis. This design provides additional evidence regarding the biological influence of HA and helps address the limited experimental data directly comparing these treatment approaches [24].

Histomorphometric analysis confirmed the histological observations. The quantitative histomorphometric findings closely paralleled the qualitative histological observations. The progressive increase in new bone area corresponded with the gradual transition from immature woven bone to more organized trabecular and lamellar bone, accompanied by increased osteocyte incorporation, active osteoblast lining, and more mature marrow spaces. This agreement between qualitative and quantitative assessments strengthens the reliability of the present findings and supports the regenerative effect of hyaluronic acid throughout the healing period. New bone area increased progressively throughout healing in all experimental groups. The findings demonstrated significant effects of healing time, treatment group, and the interaction between both variables, indicating that the regenerative effect of HA became more evident as healing progressed. Although the HA + xenograft group consistently demonstrated the highest mean bone area percentage, these differences remained statistically non-significant compared with HA alone, suggesting that both treatment modalities exhibited comparable regenerative performance within the limits of the present experimental model.

Despite the consistently higher mean percentages of newly formed bone observed in the HA + xenograft group compared with the HA group at all healing intervals, these differences did not reach statistical significance. The layered application of HA over the xenogeneic graft may also have influenced the observed biological response. In this protocol, the xenograft was placed within the extraction socket before HA was applied over its superficial surface, thereby positioning HA at the graft–tissue interface and within the healing wound environment. This approach may have provided a localized HA-rich microenvironment at the graft–tissue interface; however, the present study was not designed to isolate the effect of application sequence or to determine whether layered application provides a biological advantage over mixing HA with the graft. Therefore, this possibility should be considered hypothesis-generating and requires direct investigation in future studies comparing different application techniques.

This finding may be attributed to several factors. First, the present investigation was designed as an exploratory pilot study with a relatively small sample size, which may have limited the statistical power to detect subtle intergroup differences. Second, bone regeneration is a time-dependent process, and the six-week observation period primarily reflects early healing and remodeling rather than complete bone maturation. Therefore, the osteoconductive advantage of the xenogeneic graft may become more evident during longer healing periods. Finally, the pronounced biological activity of high-molecular-weight hyaluronic acid itself may have produced substantial regenerative effects, thereby reducing the measurable additional benefit of the xenograft within the experimental conditions of the present study. Similar observations have been reported in previous experimental and clinical investigations evaluating HA-based regenerative protocols, where improvements in quantitative outcomes did not always correspond to statistically significant differences between treatment modalities [25]. Therefore, the absence of statistical significance should not be interpreted as evidence of equivalent biological performance but rather as an indication that larger adequately powered studies are required to determine whether the observed numerical differences represent true treatment effects.

From a clinical perspective, the present findings suggest that high-molecular-weight hyaluronic acid may serve as an effective adjunctive biomaterial for enhancing extraction socket healing. The comparable regenerative outcomes observed between HA alone and HA combined with xenograft indicate that HA may represent a minimally invasive treatment option in clinical situations where placement of bone graft materials is unnecessary or contraindicated. Nevertheless, direct clinical extrapolation should be made cautiously until these findings are confirmed by adequately powered randomized clinical trials.

Several limitations should be acknowledged. First, this investigation was designed as an exploratory pilot animal study with a limited sample size, resulting in only two animals per subgroup at each healing interval. Therefore, although statistically significant differences were detected, the findings should be interpreted cautiously and validated in larger preclinical studies. Second, immunohistochemical or molecular markers of osteogenesis, such as osteocalcin, RUNX2, alkaline phosphatase, and bone morphogenetic proteins, were not evaluated. Consequently, the proposed biological mechanisms remain inferential rather than directly demonstrated. Third, collagen assessment relied on Masson’s trichrome staining, which evaluates collagen distribution and density but does not directly assess collagen fiber maturation. Finally, three-dimensional radiographic evaluation using micro-computed tomography was not performed, which could have provided additional information regarding bone volume and trabecular architecture.

Future studies should include larger sample sizes based on prospective power calculations, longer healing intervals, micro-computed tomography, immunohistochemical and molecular analyses, and comparisons between different HA formulations, including cross-linked and non-cross-linked preparations. Well-designed randomized clinical trials with histological, histomorphometric, and radiographic outcomes are also warranted to determine the clinical effectiveness of HA-based alveolar ridge preservation protocols Despite these limitations, the present study provides experimental evidence supporting the biological effectiveness of high-molecular-weight hyaluronic acid in promoting extraction socket healing and contributes to the growing body of evidence regarding biomaterial-assisted alveolar ridge preservation.

### Clinical relevance

The present findings suggest that high-molecular-weight hyaluronic acid may enhance extraction socket healing by accelerating early bone formation and improving tissue organization. Although the addition of a xenogeneic bone graft showed slightly higher regenerative outcomes, hyaluronic acid alone produced comparable healing within the study period. If confirmed by future clinical studies, hyaluronic acid may represent a simple and minimally invasive adjunct for alveolar ridge preservation following tooth extraction.

## Conclusion

Within the limitations of this exploratory pilot animal study, high-molecular-weight hyaluronic acid significantly enhanced extraction socket healing by promoting new bone formation, improving trabecular organization, and increasing collagen deposition compared with spontaneous healing. The addition of a bovine-derived xenogeneic bone graft resulted in slightly higher histological and histomorphometric values; however, these differences were not statistically significant compared with hyaluronic acid alone. These findings suggest that hyaluronic acid may represent a promising adjunctive biomaterial for alveolar ridge preservation. Nevertheless, larger preclinical studies and well-designed randomized clinical trials are required to validate these findings and determine their clinical applicability.

### Study limitations

The present study has several limitations that should be acknowledged. First, this investigation was designed as an exploratory pilot animal study with a relatively small sample size, resulting in only two animals per subgroup at each healing interval. Therefore, although statistically significant differences were observed, the findings should be interpreted cautiously and confirmed in larger adequately powered preclinical studies.

Second, the assessment was limited to histological and histomorphometric analyses without immunohistochemical or molecular evaluation of osteogenic markers, such as osteocalcin, RUNX2, or bone morphogenetic proteins, which could provide further insight into the biological mechanisms underlying bone regeneration.

Third, collagen evaluation was based on Masson’s trichrome staining, which provides information on collagen distribution and density but does not directly assess collagen maturation.

Fourth, although the histomorphometric measurements were performed by a blinded examiner using standardized image acquisition, magnification, calibration, and regions of interest, and 20% of the samples were independently re-evaluated by a second examiner, a formal statistical measure of inter-observer reliability, such as the intraclass correlation coefficient (ICC), was not calculated. Therefore, inter-observer agreement was not quantitatively assessed, and future studies should include formal reliability analysis to determine the reproducibility of the histomorphometric measurements.

Finally, three-dimensional radiographic evaluation using micro-computed tomography was not performed and may provide additional information regarding bone volume and trabecular architecture in future studies. (Table 2)

**Table 2:** ANOVA assessing the effects of treatment group and healing time on the percentage of newly formed bone area

Effect	Test	Df	*F*-value	*p*-value	Partial η²
Main effect of Group	Between-subjects	2, 21	1613.829	< 0.001*	0.994
Main effect of Time	Greenhouse–Geisser	1.447, 31.02	347.262	< 0.001*	0.943
Group × Time interaction	Greenhouse–Geisser	2.951, 31.02	87.893	< 0.001*	0.893

## Data Availability

The data that support the findings of this study are available within the article and its supplementary material.

## Declarations

### Abbreviations

HA: Hyaluronic acid.

PRF: Platelet-Rich Fibrin.

PRP: Platelet-Rich Plasma.

GBR: Guided Bone Regeneration.

BMP: Bone Morphogenetic Protein.

PDL: Periodontal Ligament.

ECM: Extracellular Matrix.

LM: Light Microscope.

H&E: Hematoxylin and Eosin.
